# Animal models for bladder cancer: The model establishment and evaluation (Review)

**DOI:** 10.3892/ol.2015.2888

**Published:** 2015-01-23

**Authors:** NING ZHANG, DONGYANG LI, JIALIANG SHAO, XIANG WANG

**Affiliations:** 1 Department of Urology, Huashan Hospital, Fudan University, Shanghai, P.R. China; 2 Fudan Institute of Urology, Huashan Hospital, Fudan University, Shanghai, P.R. China

**Keywords:** bladder cancer, animal models, orthotopic models, heterotopic models, living tissue grafting

## Abstract

Bladder cancer is the most common type of tumor in the urogenital system. Approximately 75% of patients with bladder cancer present with non-muscle-invasive cancer, which is generally treated by transurethral resection and intravesical chemotherapy. In spite of different therapeutic options, there remains a very variable risk of recurrence and progression. Novel therapeutic methods of treating bladder cancer are urgently required. The exploration and preclinical evaluation of new treatments requires an animal tumor model that mimics the human counterpart. Animal models are key in bladder cancer research and provide a bridge to the clinic. Various animal bladder cancer models have been described to date, but the tumor take rate is reported to be 30–100%. Establishment of reliable, simple, practicable and reproducible animal models remains an ongoing challenge. The present review summarizes the latest developments with regard to the establishment of animal models and tumor evaluation.

## 1. Introduction

Bladder cancer is the most common type of tumor in the urogenital system, and is the seventh most common type of cancer in males and the 17th most common type of cancer in females worldwide (1). In Europe in 2012, the age-standardized incidence rate of bladder cancer was 26.9 per 100,000 and 5.3 per 100,000 in males and females, respectively. In addition, the age-standardized mortality rate for this population was 8.5 per 100,000 among males and 1.8 per 100,000 among females (2). Approximately 75% of patients with bladder cancer present with non-muscle-invasive cancer that is either confined to the mucosa (stage Ta, carcinoma *in situ*, according to the 2009 TNM classification of urinary bladder cancer) or to the submucosa (stage T1) (3). The remaining 25% of diagnosed bladder cancers are in patients who present with muscle invasion. Despite the optimal treatments, which include immunotherapy, chemotherapy and surgery, bladder cancer has a high rate of recurrence and progression (4). Therefore, novel therapeutic methods of treating bladder cancer are urgently required. The exploration and preclinical evaluation of new treatments requires an animal tumor model that simulates the human counterpart. Various animal bladder cancer models have been described to date, but the tumor take rate is reported to be 30–100% (5). Establishment of animal models that are reliable, simple, practicable and reproducible remains an ongoing challenge. Herein, we review different types of animal models of bladder cancer, and summarize the recent developments with regard to tumor detection and quantification.

Animal models of bladder cancer can be classified as orthotopic (tumor growth occurs in the bladder) or heterotopic (tumor growth occurs outside of the bladder) tumor models, according to the location of the tumor. The models can be also divided into three categories: Xenogeneic (implantation of human bladder cancer cells into immunodeficient mice), syngeneic (implantation of murine bladder cancer cells into immunocompetent mice) and transgenic (genetic engineering/knock out mice) (5,6).

In order to provide a more reliable research basis, the models should be as close to human pathology and biochemical characteristics as possible. In evolutionary biological terms, large animals have more similarity to humans with regard to genetics and morphology (6,7). However, the most widely used animal models are rodents, in particular mice and rats (8). The studies showed that the structure and function of the rodent lower urinary tract is very similar to that of humans, exhibiting similar background gene expression during the oncogenesis of bladder cancer; therefore, rodents are suitable for the establishment of human bladder cancer models (9). In addition, rodents have the advantage that they reproduce easily in a short time period, are easy to maintain with low cost and can be manipulated genetically, thus remaining a critical tool in bladder cancer research (6).

## 2. Animal models for bladder cancer

### Induced bladder cancer models

Numerous human bladder cancers are considered to be caused by exposure to enviromental chemical carcinogens, including tobacco, aromatic amines and chlorinated hydrocarbons, among others (11). Therefore, research on the association between chemical carcinogenesis and the development of bladder cancer is indispensable. The model of bladder cancer induced by chemical carcinogens is a syngeneic model. The commonly used carcinogens in the model include N-methyl-N-nitrosourea (MNU), N-[4-(5-nitro-2-furyl)-2-thiazolyl]-formamide (FANFT) and N-butyl-N-(4-hydroxybutyl)nitrosamine (BBN) (5). MNU is a carcinogen that acts directly on the urothelium by intravesical instillation. It is a genotoxic compound, which can act as an initiator or as a promoter, and cause persistent DNA methylation (10). In orthotopic rodent models induced by chemical carcinogens, the process of tumor induction usually takes 8 weeks, and the rate of tumor take is ~70% (12). The tumors induced by BBN in mouse bladder cancer models are histologically and genetically similar to tumors identified in human bladder cancer (13). BBN can be administered either orally (in the diet) or intravesically; however, the establishment of tumor models induced by BBN takes 5–8 months (14). Therefore, BBN is not ideal for general use in bladder cancer research. FANFT is an indirect chemical carcinogen, the metabolites of which are excreted via the urine and combine with DNA of epithelial cell and then lead to gene mutation and tumorigenesis. This stimulates the bladder mucosa, and the bladder tumors induced by FANFT mostly develop into transitional cell carcinoma (TCC) after it is fed to rodents for 5 to 8 months (14). Numerous other chemicals, such as benzidine, phenacetin and 2-naphthylamine, have also been used as carcinogens in orthotopic rodent bladder models (15). Inorganic arsenic has long been known to be carcinogenic to humans, inducing carcinomas of the skin, urinary bladder, lung and possibly other tissues, following exposure through the drinking water, occupational inhalation, diet and pharmaceuticals. Arsenic-induced bladder cancer in the rat model reveals its mechanism of action, which involves the generation of the reactive metabolite, dimethylarsinic acid, which is carcinogenic to the rat urinary bladder as it causes cytotoxicity and regenerative proliferation (15). In general, the process of tumor induction takes 2–8 months, however this may vary between different carcinogens and therefore should be taken into consideration.

### Orthotopic transplantation models

This is the most widely used animal model, and can be built rapidly. Generally, the mass can be detected in growing for 1–2 weeks after the tumor cell is inoculated. Several human TCC cell lines are available commercially, including KU7, KU-19–19, T24, UM-UC1, UM-UC3 and UM-UC13, which may be instilled transurethrally to establish xenograft models (16,17). The rodent TCC cell lines MB49, MBT-2 and BTT-T739 are frequently used to produce the bladder tumors (18,19). For xenograft models, namely xenogeneic models, immunodeficient mice are required to avoid immune rejection, such as the athymic nude, severe combined immunodeficiency (SCID), and non-obese diabetic-SCID mice (20). A disadvantage of the xenograft model is that the immune response of intravesical therapy cannot be evaluated in mice with immune deficiencies. When rodents have immunocompetence, homograft models can only be used in the same strain in order to avoid rejection (6).

Urothelial cancer cells can be instilled transurethrally by using a 22- or 24-G urethral catheter. A number of factors can affect the tumor take rate, such as the bladder pretreatment, the amount of TCC cells instilled, the TCC cell concentration, the instillation volume and the tumor cell dwell time in bladder (5). As reported, increasing the amount of cancer cells increases the tumor implantation rate (21,22). An increase in the tumor cell dwell time means an increase in the duration for which the cancer cells are in contact with the bladder mucosa, resulting in an increase in the tumor inoculation rate (22,23).

The surface of the bladder mucosa is covered by a layer of glycosaminoglycan, which acts as a natural protective barrier to external substances and tumor implantation. Therefore, in order to successfully instill the tumor cells, this barrier must firstly be destroyed. Early approaches to orthotopic tumor implantation required an open surgical procedure to expose the mucosa for the tumor cells, which can lead to the development of muscle-invasive tumors or metastatic tumors or surgical complications, which subsequently affected the utility of this method (24–26). Jager et al (27) reported a novel high-precision approach. The bladder cancer cell lines were inoculated into 50 ten-week-old athymic nude mice by percutaneous injection under ultrasound guidance. The presence of a tumor in the anterior bladder wall could be detected in all animals 3 days later, by a bioluminescence imaging system. A new method of establishing transplantable orthotopic bladder tumors in mice has been reported, which involves directly scraping the urothelium with stylets, in order to mechanically disrupt the mucosa. Yang *et al* (19) reported that the bladder tumor incidence rate and the median survival time of the tumor-bearing mice were 100% and 26.69(±9.24) days, respectively. The bladder mucosa can also be abraded by cauterization with a transurethral cautery wire with an electrical current. For this method, in murine orthotopic bladder cancer models, tumor take rates of up to 100% have been reported, with no bladder perforations (18,28,29). Additional bladder pretreatments include the instilling of acid, trypsin and poly-l-lysine, among others (30–32). Watanabe *et al* (31) reported the use of 0.1 ml 0.2% intravesical trypsin prior to treatment for 30 min, and mechanical bladder injury immediately before cancer cells were instilled for 3 h. The tumor take rate was 90% by microscopic examination of the bladder 10 days following implantation, however, the procedure-related death rate was ~30%. An additional study reported a tumor take rate of 94% after instilling 0.1 mL of 0.1mg/ml poly-l-lysine for 20 min prior to cancer cell implantation (30).

### Spontaneous bladder cancer models

Naturally occurring cancer models have markedly greater similarity to their counterparts in humans than many currently used experimentally induced tumor models and thus, may accurately reflect the mechanism and process of tumorigenesis, as cancer in animals may mimic specific forms of human cancer with regard to histopathological characteristics, cellular and molecular features, biological behavior and response to therapy (33). Knapp et al (34) conducted an in-depth and extensive study on spontaneous bladder TCC of 102 dogs, and provided a favorable experimental basis to study the tumorigenic mechanism and evaluate new strategies for cancer therapy. However, spontaneous bladder cancers in mice are rare, the establishment of models is complicated and time-consuming, and the repeatability of models cannot be guaranteed; therefore, this not a commonly used model (35).

### Genetic engineering models

Activation of oncogenes such as Ha-ras or alteration in the suppressor genes RB1 and p53 in the urothelium is considered critical for the development of urothelial tumors (36,37). Genetically engineered mouse models have been developed, which may more closely mimic the human disease at the molecular level (38). Transgenic mice which carry certain genes and certain mice strains, that exhibit knocked out tumor suppressor genes, have been shown to exhibit increased carcinogen susceptibility (39,40). A study showed that mice deficient in both pRB and p53 are highly susceptible to carcinogen exposure; such mice developed invasive carcinomas that resembled human bladder cancer (41). In addition, transgenic mice with compromised immune systems have been developed. Mice in which genes, such as interferon-γ and interleukin 17, 12 and 23, among others, are knocked out, are being used to determine how different components of the immune system either promote or inhibit the development of bladder tumors (42–44). Transgenic mice are a particular type of syngeneic models that are genetically modified to study the importance of a particular gene in cancer development and progression. Knockout mice are genetically modified mice, and can be used to study the effect of the deficiency of a specific gene (38). With regard to the mechanism of RNA interference, the use of intravesical small interfering RNA, which can silence abnormally upregulated genes in cancer, has been demonstrated to be effective in the treatment of bladder cancer (12). However, genetic engineering models also have several limitations. Firstly, tumors that develop in these models tend to be less heterogeneous than human bladder tumors, which may influence their progression and metastasis. Secondly, the cells derived from an animal model may not accurately reflect the ones derived from the corresponding human condition (38).

### Heterotopic animal models

Heterotopic animal models involve the growth of a mass in a location different from its target organs, generally using subcutaneous inoculation or subrenal capsule xenografts. In both syngeneic and xenogeneic models, tumors may grow in heterotopic or orthotopic sites. For the subcutaneous inoculation, in which the tumor cells are injected into the subcutis of immunodeficient mice, the process of oncogenesis is easily assessed by using palpation of the skin and measurement with a caliper (45). Previous studies have revealed that subcutaneous inoculation of murine MB49 or 253JB-V bladder cancer cell lines did not develop metastasis to lymph nodes and lung, and only tumors growing orthotopically in the bladder did (45,46). For the subrenal capsule xenograft, in which the living human cancer tissues are generally grafted beneath the renal capsule of SCID mice through open surgery, the tumor take rate is evidently higher than that of tumors from subcutaneous xenografts of cancer tissues, due to the abundant blood supply of the renal capsule (47,48). Due to the existence of living cancer tissue stroma, which supports the growth of tumor, this method can simulate the original microenvironment that is suitable for tumor growth (46). In recent years, increasing attention has been paid to the establishment of models by living tissue grafts. Certain preclinical experimental xenograft models from fresh tumor tissue grafts have been developed for rare cancers, such as neuroendocrine bladder cancer, which provide novel tools for the discovery of drug and diagnostic targets (49).

Other heterotopic models include the inoculation of cancer cell lines into the tail vein or the left ventricle of the heart to develop tumors in the lung or bone. These rodent bladder cancer models have been widely used to evaluate the process of tumor metastasis and colonization (50,51).

## 3. Evaluation of animal models for bladder cancer

As the tumor take rate of animal models is usually less than 100%, and the formation of a mass cannot be observed by the naked eye in orthotopic or subrenal capsule animal models, it is important to monitor the tumor formation and confirm the origin of the tumor cells, in order to evaluate whether the cancer cells have changed in the process of growth (5).

Physical examination includes the direct palpation of a subcutaneous or bladder mass, or bladder inspection by a transurethral mini cystoscope (52). Furthermore, hematuria is also a indicator for the formation of tumor. However, hematuria and a palpable bladder mass may indicate a late-stage tumor.

With regard to the non-invasive methods for evaluating tumor growth, in vivo imaging systems are widely used. Ultrasound (US) examination of the urinary bladder is often used to monitor tumor growth. High-frequency intravesical US has recently been utilized in the orthotopic implanted mouse model for bladder cancer, and was reported to exhibit high sensitivity (53). Another study showed that, using targeted contrast-enhanced micro-US imaging, investigators are able to detect and monitor vascular changes in mouse orthotopic bladder tumor models (54). Magnetic resonance imaging has been used for early tumor detection, as tumor growth may be identified 14 days after tumor implantation in mice with no clinical signs of disease, as well as for monitoring tumor growth and the therapy-induced changes in murine bladder tumor models, particularly in the subrenal capsule xenograft or intravesical tumor models (55). A flat panel detector-based cone beam computed tomography system has been reported to be used for the early detection and measurement of urothelial tumors, including the tumor position, volume and growth (56). Bioluminescent imaging can be used for real-time sensitive cancer cell tracking during tumor growth, progression and metastasis, and can also be used to measure the efficacy of tumor therapy (57).

In addition, for the xenogeneic models, hematoxylin and eosin staining should be performed to confirm the tumor mass still retains the major histological features of the original tumor (48). Immunohistochemistry is conducted to confirm the human origin of the tumor cells and development in xenografts of murine supportive stroma (49). By indirect immunological detection, the reaction between monoclonal antibody BDI-1 and human TCC tissues is strongly positive, while that for normal human and murine tissues is negative (58).

## 4. Discussion and conclusions

As the most common malignancy of the urinary tract, bladder cancer has become a significant public health issue worldwide, particularly given the increasing cost for treating either non-muscle-invasive or muscle invasive disease. Therefore, it is essential to intensify bladder cancer research. The development of reliable, stable and simple animal models plays an important role in the research.

Ideally, animal models for urinary bladder carcinogenesis must be similar to human bladder cancer in their histology, biochemical properties, molecular and genetic characteristics, natural history and biological behavior, when evaluating future therapies. Compared with the ectopic tumor models, orthotopic tumor models simulate the natural environment of bladder cancer, demonstrating intact pathological and immunological responses. The orthotopic tumor often appears muscle-invasive or metastatic, which may be used to investigate tumor invasion and metastasis. Each type of rodent model for bladder carcinogenesis studies has its own advantages and disadvantages, and the aims of the research will dictate which type of model is most suitable (12).

Conventional treatments, including surgery, intravesical therapy and BCG instillations, decrease the recurrence rate of bladder cancer, but are associated with side effects, such as sepsis and cystitis, as well as frequent failures, which include tumor recurrence or progression (11). Therefore, there is a clear requirement for the development of highly effective targeted therapies with limited side effects. Successful preclinical models of bladder cancer may be used to investigate various aspects of tumor progression, and provide a platform for developing novel therapeutic regimens and confirming the therapeutic potential of new promising drug candidates, with the possibility of patient-tailored therapies (59).

Studies using patient-derived, clinically highly relevant cancer xenograft models appear to be more promising as the properties of human cancer are better reflected using tumor models derived from the patient’s cancer tissue (49,59). Through the research on xenograft models from fresh tumor tissue grafts, it is possible to establish the transplantable cancer tissue lines from a variety of low and higher grade malignancies. Therefore, we can improve the predictive value of preclinical drug efficacy evaluation of new or existing anticancer drugs by using the xenograft models. We can provide cancer studies with a highly humanized in vivo cancer model and develop a translational link between the laboratory and the clinic. We can also provide a service for identifying which drug regimen, from a group of selected drug regimens, has potentially the highest efficacy for an individual patient and can be utilized for the patient’s personalized chemotherapy.
